# Insights from the reconstitution of the divergent outer kinetochore of *Drosophila melanogaster*

**DOI:** 10.1098/rsob.150236

**Published:** 2016-02-24

**Authors:** Yahui Liu, Arsen Petrovic, Pascaline Rombaut, Shyamal Mosalaganti, Jenny Keller, Stefan Raunser, Franz Herzog, Andrea Musacchio

**Affiliations:** 1 Department of Mechanistic Cell Biology, Max Planck Institute of Molecular Physiology, Otto-Hahn-Straße 11, 44227 Dortmund, Germany; 2 Department of Structural Biochemistry, Max Planck Institute of Molecular Physiology, Otto-Hahn-Straße 11, 44227 Dortmund, Germany; 3 Gene Center Munich, Ludwig-Maximilians-Universität München, Feodor-Lynen-Strasse 25, 81377 Munich, Germany; 4 Centre for Medical Biotechnology, Faculty of Biology, University Duisburg-Essen, Universitätsstraße, 45141 Essen, Germany

**Keywords:** kinetochore, centromere, KMN network, Mis12, Spc105, Ndc80

## Abstract

Accurate chromosome segregation during mitosis and meiosis is crucial for cellular and organismal viability. Kinetochores connect chromosomes with spindle microtubules and are essential for chromosome segregation. These large protein scaffolds emerge from the centromere, a specialized region of the chromosome enriched with the histone H3 variant CENP-A. In most eukaryotes, the kinetochore core consists of the centromere-proximal constitutive centromere-associated network (CCAN), which binds CENP-A and contains 16 subunits, and of the centromere-distal Knl1 complex, Mis12 complex, Ndc80 complex (KMN) network, which binds microtubules and contains 10 subunits. In the fruitfly, *Drosophila melanogaster,* the kinetochore underwent remarkable simplifications. All CCAN subunits, with the exception of centromeric protein C (CENP-C), and two KMN subunits, Dsn1 and Zwint, cannot be identified in this organism. In addition, two paralogues of the KMN subunit Nnf1 (Nnf1a and Nnf1b) are present. Finally, the Spc105R subunit, homologous to human Knl1/CASC5, underwent considerable sequence changes in comparison with other organisms. We combined biochemical reconstitution with biophysical and structural methods to investigate how these changes reflect on the organization of the *Drosophila* KMN network. We demonstrate that the Nnf1a and Nnf1b paralogues are subunits of distinct complexes, both of which interact directly with Spc105R and with CENP-C, for the latter of which we identify a binding site on the Mis12 subunit. Our studies shed light on the structural and functional organization of a highly divergent kinetochore particle.

## 1. Introduction

Accurate chromosome segregation in dividing cells is of utmost importance for the propagation of unicellular organisms, for organismal development and for sexual reproduction [1]. Perturbations of this process have been associated with congenital diseases, premature ageing and cellular transformation [2].

**Figure 1.**
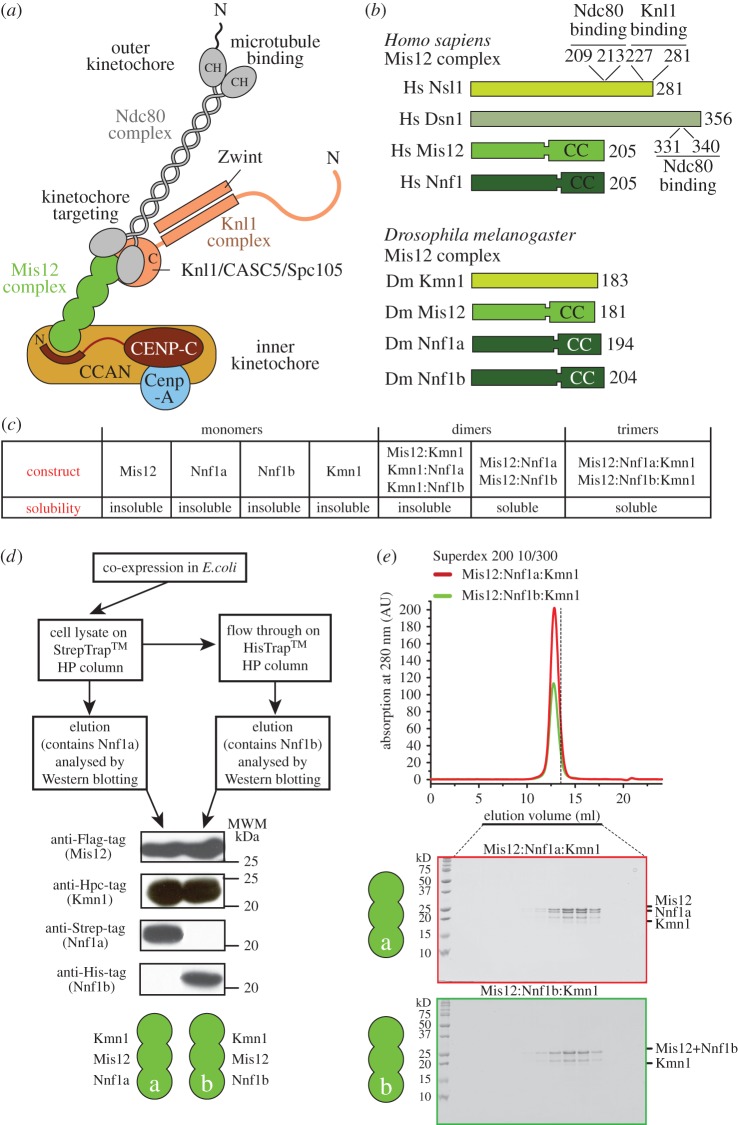
Two Mis12 complexes in *Drosophila melanogaster*. (*a*) Schematic of the human kinetochore. Orthologues of the indicated subunits and complexes are generally conserved in evolution, and are for instance also identified in *S. cerevisiae*. (*b*) A presentation of the constitutive subunits of the Mis12 complex in humans and in *Drosophila*. Segments identified for their ability to interact with Knl1 or Ndc80 complex subunits [6,7] are indicated. (*c*) Summary of expression experiments. ‘Soluble’ or ‘insoluble’ indicates that the protein could/could not be identified in the bacterial cell lysate. (*d*) A strategy for determining whether Nnf1a and Nnf1b are part of the same or different complexes. (*e*) Size-exclusion chromatography (SEC) experiment on the DmMis12a and DmMis12b complex showing the two complexes elute in a single peak and appear monodisperse. The vertical dashed bar is a reference indicating the elution volume of the dimeric constructs shown in electronic supplementary material, figure S1A.

The mitotic spindle, a complex structure made of microtubules, microtubule-associated proteins and molecular motors, is devoted to chromosome capture and segregation [1]. Microtubules capture chromosomes at specialized and evolutionarily conserved structures named kinetochores [3,4]. Kinetochores are multi-protein assemblies that are built on a specialized chromatin domain called the centromere [5]. The crucial and universal feature that distinguishes the centromere from any other chromatin domain on chromosomes is the enrichment of a variant of histone H3 named CENP-A (CID in *Drosophila melanogaster*; figure 1*a*) [5]. In most organisms, this histone variant recruits the components of a constitutive centromere-associated network (CCAN), a group of approximately 16 proteins organized in different subcomplexes [8–12]. The CCAN, in turn, recruits the components of a 10-subunit complex named the KMN network (for Knl1 complex, Mis12 complex, Ndc80 complex, the three subcomplexes of which the KMN network is composed) [13]. Within the KMN network, the Ndc80 complex (Ndc80-C) has been implicated as the microtubule receptor at the kinetochore [14,15]. The Knl1 complex (Knl1-C), on the other hand, has been implicated in the coordination of the spindle assembly checkpoint, a signalling mechanism that prevents premature mitotic exit in the presence of unattached or incorrectly attached kinetochores [16]. Finally, the Mis12 complex (Mis12-C, also known as the MIND complex in *Saccharomyces cerevisiae*) acts as a ‘hub’ that interacts with all other KMN complexes and that also mediates the interaction with the inner kinetochore CCAN subunits [6,17–31]. Furthermore, the Mis12 complex may increase the binding affinity of the Ndc80 complex for microtubules, possibly through an allosteric mechanism [32].

In certain organisms, including *Drosophila melanogaster* and *Caenorhabditis elegans*, most CCAN subunits cannot be identified, suggesting that these kinetochores underwent significant structural simplifications in the course of evolution [4,13,20,33–36]. To date, the only residual CCAN subunit to be clearly recognizable in these organisms is CENP-C [37–40]. CENP-C, which is the largest CCAN subunit, has been shown to act as a linker between CENP-A in the centromeric chromatin and the Mis12-C in the outer kinetochore [19–21,23,41–44]. In organisms that retained CCAN, CENP-C also contains binding sites for other CCAN subunits, including the CENP-HIKM and CENP-NL subcomplexes [45–49]. Finally, CENP-C has been shown to participate in the cell-cycle-dependent deposition of new CENP-A required to re-establish the CENP-A pool after its halving during chromosome replication [50–60].

Besides the loss of most CCAN subunits in the inner kinetochore, in *D. melanogaster* additional evolutionary changes affected the composition of the outer kinetochore, and in particular of the Mis12-C complex. These changes include the apparent loss of the Dsn1 subunit, the appearance of two paralogues of the Nnf1 subunit (Nnf1a and Nnf1b, also named Nnf1R-1 and Nnf1R-2), and the loss of the Zwint subunit in the Knl1-C, which therefore consists exclusively of the Spc105R subunit (Spc105-related, homologous to human Knl1/Blinkin/CASC5 and indicated here as Spc105R^Knl1^) [17,26,31,33,34,61–63]. How these changes affect the overall organization and stability of the outer kinetochore and of its interactions with CENP-C is currently unclear. Here, we used biochemical reconstitution and biophysical characterization as an entry point to characterize the outer kinetochore of *D. melanogaster* and its interaction with CENP-C. We report the main conclusions of our effort.

## 2. Results and discussion

### 2.1. Reconstitution of two related Mis12 complexes in *Drosophila melanogaster*

To gain insights into the organization of the DmMis12 complex, we expressed recombinant versions of its subunits (figure 1*b*) or their combination, as summarized in figure 1*c*. Mis12, Nnf1a, Nnf1b and Kmn1 (the latter indicated as Kmn1^Nsl1^ to remind readers that it is the Nsl1 orthologue) were all insoluble when expressed in isolation in *Escherichia coli* (not shown). Co-expression of different combinations of two subunits with the pST44 vector [64] resulted in soluble complexes of Mis12 with Nnf1a or Nnf1b, whereas binary combinations containing Kmn1^Nsl1^ were insoluble (figure 1*c*; electronic supplementary material, figure S1*a*; some data not shown). Overall, these results suggest that Mis12 and Nnf1 can form a stable pair within the *Drosophila* Mis12 complex, in line with previous observations [18,25,28,63,65]. Solubilization of Kmn1^Nsl1^ was only observed when it was co-expressed in combination with Mis12 and Nnf1a or Nnf1b (figure 1*c*).

The Nnf1a and Nnf1b paralogues have been previously shown to be functionally redundant, but their developmental expression patterns are not identical [34,63]. It is unclear if these proteins are incorporated in the same complex or in separate complexes. The question is particularly relevant in the specific case of the *Drosophila* Mis12-C, because no Dsn1 has been identified in this organism, suggesting that Mis12-C might have a different composition. To address this question, we co-expressed Mis12, Nnf1a, Nnf1b and Kmn1^Nsl1^, each fused to a distinct tag, in *E. coli* (figure 1*d*). Cleared cell lysates were incubated, in consecutive steps, with affinity resins designed to interact with the affinity tags of Nnf1a (Strep tag) and Nnf1b (polyhistidine), and after elution each bound fraction was analysed by Western blotting (figure 1*d*). This showed that Nnf1a and Nnf1b are both able to bind Mis12 and Kmn1^Nsl1^, but do not appear to interact with each other in the same complex.

We reconstituted the Mis12a and Mis12b complexes by bacterial co-expression and purified them to homogeneity (see Methods). Separation of these complexes by size-exclusion chromatography (SEC, which separates based on shape and molecular mass) demonstrated that both complexes are monodisperse and that they elute essentially identically, suggesting similar shape and overall mass (figure 1*e*). Overall, these data demonstrate that Nnf1a and Nnf1b form distinct and stoichiometric complexes with Kmn1^Nsl1^ and Mis12, which we define as the DmMis12a and DmMis12b complexes, respectively.

### 2.2. Characterization of the DmMis12a and DmMis12b complexes

**Figure 2.**
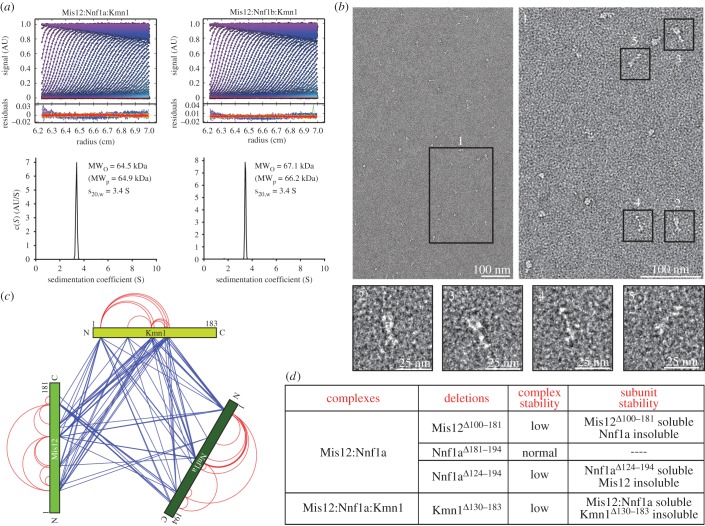
Biophysical analysis of the DmMis12 complexes. (*a*) Sedimentation velocity absorbance profiles of the DmMis12a and DmMis12b complexes, with residuals of the fit showing the deviation of the c(*S*) model from the observed signals; the best-fit continuous-size c(*S*) distribution of the DmMis12a and DmMis12b complexes is shown in the bottom part of the panel. (*b*) Representative negative stain EM images of the DmMis12a complex. Scale bars are indicated. (*c*) Cross-linking-mass spectrometry (XL-MS) analysis of the DmMis12a complex. Blue and red lines indicate inter- and intramolecular cross-links, respectively. (*d*) Summary of expression experiments with different deletion mutants of the subunits of the DmMis12 complex.

By analytical ultracentrifugation (AUC) sedimentation velocity experiments, we observed molecular masses of the DmMis12a and DmMis12b complexes of 64.5 and 67.1 kDa, respectively (figure 2*a* and table 1). These values are in excellent agreement with the predicted molecular masses if each subunit was represented in a single copy (table 1). Frictional ratios (*f*/*f*_o_) of 1.7 indicate that both complexes are elongated.

**Table 1.** Summary of sedimentation velocity experiments. All predicted molecular masses assumed each subunit was present in a single copy.

complexes	frictional ratio	observed molecular mass (kDa)	predicted molecular mass (kDa)	S (20,w)
Mis12:Nnf1a:Kmn1	1.7	64.5	64.9	3.4
Mis12:Nnf1b:Kmn1	1.7	67.1	66.2	3.4
Mis12:Nnf1a:Kmn1:Cenp-C^1–105^	1.9	76.4	76.8	3.4
Mis12:Nnf1b:Kmn1:Cenp-C^1–105^	1.9	75.6	78.0	3.4
Mis12:Nnf1a:Kmn1:Spc105R^1707–1960^	1.7	93.3	95.2	4.3
Mis12:Nnf1b:Kmn1:Spc105R^1707–1960^	1.6	95.7	96.4	4.8
Mis12:Nnf1b:Kmn1-Spc105R^1707–1960^-Cenp-C^1–105^	1.8	106.5	109.3	4.4

This was confirmed by negative-stain electron microscopy (EM) experiments on the DmMis12a complex (figure 2*b*). In each field of view, the majority of single particles appeared elongated, with a thicker end and an overall length of approximately 20 nm. The appearance of the DmMis12a complex is largely comparable to that of the previously observed human and budding yeast complexes [6,24,25,65]. Thus, loss of Dsn1 does not dramatically alter the structure of the DmMis12 complex. However, despite high purity, compositional homogeneity and monodispersity of the Mis12 emerging from SEC experiments (figure 1*e*), we observed more structural heterogeneity of the complex by negative stain EM (figure 2*b*) than previously observed with the human complex [6,24], complicating the calculation of class averages. In summary, the EM and AUC analyses indicated that the DmMis12 complex has an elongated appearance, a feature previously observed with the human and yeast complexes [6,19,24,25,65].

To gain additional insights into the organization of the DmMis12a and DmMis12b complexes, we resorted to chemical cross-linking with the bi-functional reagent BS2G (bis[sulfo-succinimidyl]glutarate), which cross-links the primary amines of lysine side chains within a distance compatible with the length of the cross-linker (7.7 Å) (electronic supplementary material, figure S1*b*), followed by protease digestion and mass spectrometry (XL-MS) [66]. The analysis revealed a very extensive network of interactions between the Mis12 and Nnf1a or Nnf1b subunits, extending all along their sequences (figure 2*c*; electronic supplementary material, figure S1*c*). Both subunits also become extensively cross-linked to the N-terminal region of Kmn1^Nsl1^, extending approximately to residue 120. However, residues 130–183 in the C-terminal region were required for a stable interaction of Kmn1^Nsl1^ with the rest of the DmMis12a complex, because their deletion (Kmn1^Δ130–183^) generated an unstable mutant that failed to be incorporated in a complex with Nnf1a and Mis12 (figure 2*d*). Large C-terminal deletions of Mis12 and Nnf1a also strongly reduced the stability of the binary Mis12:Nnf1a complex (figure 2*d*; some data not shown).

### 2.3. DmMis12-C interacts directly with CENP-C

**Figure 3.**
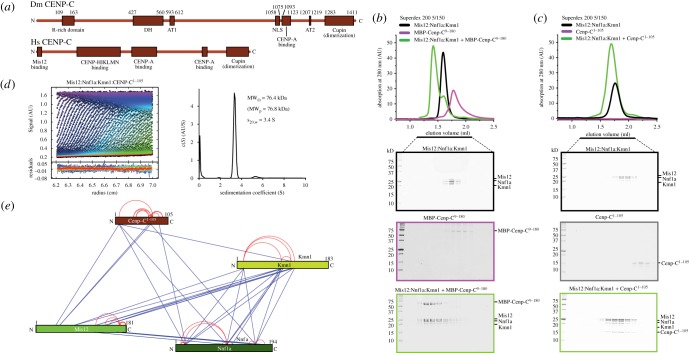
The DmMis12 complex binds the N-terminal region of CENP-C. **(***a*) Schematic comparison of the domain structure of CENP-C in *Drosophila* and humans. Domains in the *Drosophila* sequence are as follow: R-rich, arginine-rich; DH, drosophilid Cenp-C homologues; AT1 and AT2, AT hooks; NLS, nuclear localization signal; CENP-A binding motif, also known as the CENP-C motif; Cupin, a dimerization domain near the C-terminal region (C-term). For more detail, see [38]. Human CENP-C contains an N-terminal Mis12 binding domain [19,20,28], a domain for binding to the CENP-HIKM and CENP-NL complexes [46,49], and domains related to those present in *Drosophila*. (*b*) Analytical size-exclusion chromatography shows that the DmMis12a complex binds directly to MBP-CENP-C^9–180^. (*c*) Analytical size-exclusion chromatography shows that the DmMis12a complex binds directly to CENP-C^1–105^. (*d*) Sedimentation velocity absorbance profiles of the DmMis12a and DmMis12b complexes, with residuals of the fit showing the deviation of the c(*S*) model from the observed signals; the best-fit continuous-size c(*S*) distribution of the DmMis12a and DmMis12b complexes is shown on the right-hand side of the panel. (*e*) Cross-linking-mass spectrometry (XL-MS) analysis of the DmMis12a complex. Blue and red lines indicate inter- and intramolecular cross-links, respectively.

CENP-C, a subunit of the CCAN complex, interacts directly with the specialized CENP-A nucleosome in the centromere chromatin underlying the kinetochore (see Introduction). Comparison of the overall organization of CENP-C in *Drosophila melanogaster* and in other metazoans reveals remarkable differences (figure 3*a*). For instance, DmCENP-C is approximately 500 residues longer than HsCENP-C [38]. Within its N-terminal half, DmCENP-C sequence contains two regions, the arginine-rich (R-rich) domain and the drosophilids CENP-C homology (DH) domain [38], that cannot be detected in the human sequence. It also contains two predicted AT-hooks domain (AT1 and AT2), which may mediate interactions with DNA [38]. The function of all these domains unique to the *Drosophila* sequence is currently unclear. In humans, a region of CENP-C also located in the N-terminal half of the protein has been recently implicated in binding to CCAN subunits such as CENP-H, CENP-I and others (figure 3*a*) [46,49]. Thus, divergence in the N-terminal region of CENP-C may reflect the specific evolutionary history of *Drosophila* that led to the loss of other CCAN subunits. On the other hand, the C-terminal region of DmCENP-C, containing a CENP-C motif implicated in CENP-A binding and a dimerization domain [38,67], is related to metazoans' (figure 3*a*).

In previous studies, we and others demonstrated that Mis12-C binds directly to CENP-C in *Drosophila*, budding yeast and humans [19,20,28]. In humans, as little as approximately 20 residues at the N-terminus of CENP-C are sufficient to generate a relatively tight binding interaction with Mis12-C, whereas longer CENP-C segments bind more tightly [19]. Similar conclusions emerged from studies in *S. cerevisiae* [28]. An alignment of the N-terminal region of CENP-C in drosophilids, yeasts and vertebrates failed to reveal strictly conserved features, although a possible fuzzy pattern consisting of a stretch of positive charges followed by hydrophobic stretches might be envisioned (electronic supplementary material, figure S2).

Because the domain of DmCENP-C interacting with the Mis12 complex has not been mapped in detail, we tested binding of the DmMis12a complex to a fusion protein of maltose binding protein (MBP) with residues 9–180 of CENP-C (CENP-C^9–180^) in an SEC experiment (figure 3*b*). A clear shift in the elution pattern of both species was indicative of a tight interaction. Essentially identical results were obtained with DmMis12b complex (electronic supplementary material, figure S3*a*). Residues 1–8 of DmCENP-C are not conserved in other drosophilids, but conservation increases significantly in regions immediately C-terminal to this non-conserved region (electronic supplementary material, figure S2). Indeed, larger N-terminal deletions (DmCENP-C^36–180^) prevented an interaction with both the Mis12a and Mis12b complexes (electronic supplementary material, figures S3*b*,*c*), indicating that residues 9–35 contain essential interaction determinants.

We then tested the effects of C-terminal deletions from the DmCENP-C N-terminal region. A construct corresponding to DmCENP-C^1–105^ (devoid of affinity tags) interacted with the DmMis12a and DmMis12b complexes stoichiometrically (figure 3*c*; electronic supplementary material, figure S3*d*), and so did an even shorter deletion mutant, DmCENP-C^9–71^ (also devoid of tags; electronic supplementary material, figure S3*e*,*f*). Collectively, these results demonstrate that the Mis12 complex binds directly to the N-terminal region of CENP-C in *Drosophila*, similarly to what was previously observed in humans and yeast [19,28], and despite the very modest sequence identity in the CENP-C N-terminal region across species. The DmMis12a:DmCENP-C^1–105^ complex was monodisperse in SEC runs and sedimented essentially as a single peak in sedimentation velocity experiments (figure 3*d*), with a predicted molecular mass of 76.4 (table 1), indicating that the Mis12a complex and CENP-C interact with 1 : 1 stoichiometry. Essentially identical results were obtained with the Mis12b:CENP-C complex (table 1 and electronic supplementary material, figure S4*a*). XL–MS experiments confirmed an interaction of CENP-C^1–105^ with the Mis12 subunit, but also identified additional potential contacts with Nnf1 and Kmn1^Nsl1^ (figure 3*e* and electronic supplementary material, figure S4*b*).

### 2.4. A CENP-C binding site on the Mis12 subunit of the Mis12 complex

The determinants of the Mis12 complex required to interact with CENP-C are unknown, although a requirement for the Nnf1 subunit *in vitro* has been described [20]. In our attempts (until now unsuccessful; data not shown) to crystallize the *D. melanogaster* Mis12 complex, we generated a version of the Mis12a complex in which the first 15 residues of the Mis12 subunit had been deleted. Unexpectedly, the deletion mutant failed to bind CENP-C^1–105^ (electronic supplementary material, figure S5*a*).

**Figure 4.**
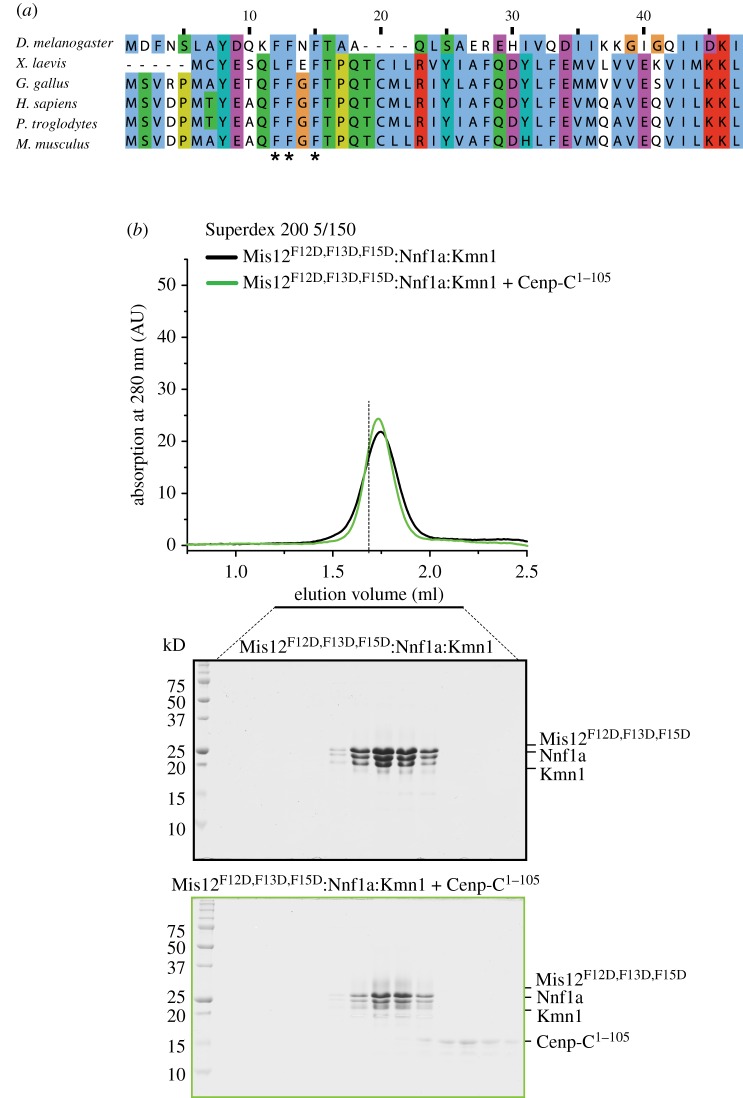
A CENP-C binding region in the Mis12 subunit. (*a*) Sequence alignment of the N-terminal region of the Mis12 subunit of the Mis12 complex. The positions of three phenylalanine (F) residues that were mutated to Asp are indicated by asterisks. (*b*) Analytical size-exclusion chromatography shows that the CENP-C^1–105^ is unable to interact with the mutant Mis12 complex. The vertical dashed bar is a reference indicating the peak elution volume of the tetrameric MIs12:Nnf1a:Kmn1:CENP-C^1–105^ complex whose elution is shown in figure 3*c*.

The sequence of the first 15 residues of the Mis12 subunit is evolutionarily conserved (figure 4*a*). Because removal of this region does not appear to be grossly detrimental to the stability of the Mis12 complex, we tested the role of three conserved phenylalanine (F) residues, F12, F13 and F15, in the interaction with CENP-C^1–105^. A DmMis12a complex containing mutations F12D, F13D and F15D in the Mis12 subunit was monodisperse, as judged by its SEC elution profile (figure 4*b*). In agreement with a role of the N-terminal region of Mis12 in CENP-C binding, the mutant was unable to interact with CENP-C^1–105^ in a SEC co-elution experiment, indicating that the mutations disrupt the interaction of Mis12 with CENP-C (figure 4*b*). Essentially identical results were obtained with a DmMis12b complex expressing mutations F12D, F13D and F15D (electronic supplementary material, figure S5*b*). Thus, our results implicate the N-terminal region of the Mis12 subunit as a necessary determinant of the interaction of the Mis12 complex with CENP-C.

### 2.5. The interaction of the Mis12 complex with Spc105R

**Figure 5.**
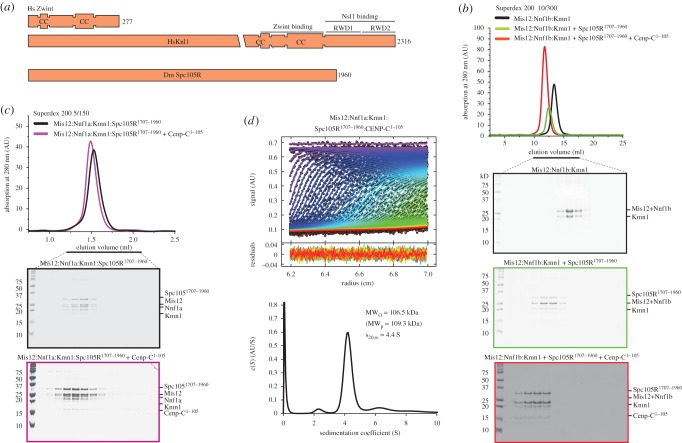
Interaction of the Mis12 complex with the C-terminal region of DmSpc105. (*a*) Schematic comparison of the domain structure of Spc105R1^Knl1^ in *Drosophila* and of its human homologue Knl1/CASC5. The C-terminal region of Knl1/CASC contains a coiled-coil domain that has been implicated in a direct interaction with Zwint, a coiled-coil protein that has not been identified in *Drosophila*. It also contains two consecutive RWD domains implicated in a direct interaction with the Nsl1 subunit of the human Mis12 complex [24]. (*b*) Size-exclusion chromatography analysis of the indicated complexes demonstrates that Spc105R^1707–1960^ and CENP-C^1–105^ enter a single complex with the Mis12b complex. (*c*) The Mis12a:Spc105R^1707–1960^ complex binds CENP-C^1–105^. (*d*) AUC sedimentation velocity analysis of Mis12a:Spc105R^1707–1960^:CENP-C^1–105^ complex.

Another interesting difference between the KMN network in *D. melanogaster* and other eukaryotes lies in the Knl1 complex. One of the two subunits of the complex, Zwint, has not been identified in *D. melanogaster* (figure 5*a*). Conversely, DmSpc105R^Knl1^, related to the Knl1/CASC5 subunit, is shorter than in humans. Previously, it has been shown that the C-terminal region of human Knl1 contains two consecutive RWD (RING finger, WD repeat, DEAD-like helicases) domains preceded by a coiled-coil region. The latter mediates the interaction with Zwint, which is also a coiled-coil protein, whereas the former mediate binding to the C-terminal region of the Nsl1 subunit of the human Mis12 complex, homologous to Kmn1 in *D. melanogaster* [6,24,27] (figure 5*a*).

None of these features is evident in DmSpc105R^Knl1^. First, program COILS [68] only identifies a short sequence (residues 1850–1890) with (limited) potential for forming a coiled-coil, instead of the approximately 200-residue coiled-coil domain identified in the human protein. Second, there is no evidence that the C-terminal region of DmSpc105R^Knl1^ might contain RWD domains like the human counterpart Knl1/CASC5. For instance, BLAST (http://blast.ncbi.nlm.nih.gov/Blast.cgi) searches with the last approximately 200 residues of DmSpc105R^Knl1^ fail to detect homologous proteins beyond drosophilids (not shown). Additionally, three-dimensional modelling with the Phyre2 server [69] failed to identify structural homology of the C-terminal region of DmSpc105R^Knl1^ with structures deposited in the protein data bank, which include several structures of RWD domains, including those present in Knl1/CASC5 [24] (not shown). Nevertheless, secondary structure prediction servers, including JPRED4 (http://www.compbio.dundee.ac.uk/jpred/index_up.html) and PSIPRED (http://bioinf.cs.ucl.ac.uk/psipred/) [70,71], identify a succession of secondary structure elements for residues 1850–1960 of DmSpc105R^Knl1^ that is, in principle, compatible with the presence of an RWD domain (data not shown). Thus, the detailed structural organization of the C-terminal region of DmSpc105R^Knl1^ remains uncertain. Despite possible evolutionary changes, however, previous evidence demonstrated that an approximately 600-residue construct containing the C-terminal region of DmSpc105R^Knl1^ can interact with Kmn1^Nsl1^ in a yeast two-hybrid (Y2H) experiment [17]. This suggests that the C-terminal regions of the human and fly sequences are, if not evolutionary conserved, at least functionally related.

To shed light on the interaction of DmSpc105R^Knl1^ with the Mis12 complex, we co-expressed several recombinant segments encompassing the C-terminal region of Spc105R^Knl1^ with the Mis12a or Mis12b complexes. Constructs approximately encompassing the predicted coiled-coil region (comprised in the segment 1852–1889), including Spc105R^1707–1882^ and Spc105R^1707–1890^, were insoluble. Constructs containing the C-terminal region downstream from the predicted coiled-coil, including Spc105R^1887–1960^, Spc105R^1875–1960^ and Spc105R^1890–1960^, were insoluble. Finally, constructs containing the predicted coiled-coil and the C-terminal region, including Spc105R^1847–1960^ and Spc105R^1810–1960^, were also insoluble. The only segment of Spc105R^Knl1^ that could be co-expressed in a soluble form with the Mis12a and Mis12b complexes was Spc105R^1707–1960^. In both cases, an apparently monodisperse and stoichiometric complex formed (figure 5*b*,*c*), whose behaviour in AUC sedimentation velocity experiments predicted a Mis12 complex:Spc105R^1707–1890^ stoichiometry of 1 : 1 (table 1; electronic supplementary material, figure S6*a*,*b*). Both the Mis12a:Spc105R^1707–1890^ and the Mis12b:Spc105R^1707–1890^ complexes further interacted with CENP-C^1–105^ in single monodisperse complexes (figure 5*b*,*c*). In agreement with this observation, AUC sedimentation velocity analysis of the Mis12a:Spc105^1707–1960^:CENP-C^1–105^ complex revealed a stable 1 : 1:1 assembly (table 1 and figure 5*d*).

### 2.6. Conclusion

Owing to the considerable array of interactions it mediates, the Mis12 complex is viewed as a ‘hub’ of kinetochore assembly and function. Biochemical reconstitution of the yeast and human Mis12 complexes has shed considerable light on their organization, revealing a conserved set of intra- and intercomplex interactions [6,7,24,28,65]. A detailed, high-resolution structural characterization of the Mis12 complex, however, has been missing, possibly because of the inherent flexibility of some of its domains.

Our work on the *Drosophila* Mis12 complex was motivated by its considerable simplification in comparison with its counterparts in other organisms, with one of the four subunits, Dsn1, having apparently disappeared from the *Drosophila* genome. Furthermore, because CCAN subunits are absent in *Drosophila* (with the exception of CENP-C), it may be surmised that the *Drosophila* Mis12 complex does not require stabilization through additional protein–protein interactions at the kinetochore. By way of example, the yeast Mis12/MIND complex was found to interact with the COMA complex, consisting of the Ctf19, Okp1, Mcm21 and Ame1 subunits (and homologous to CCAN subunits CENP-O, CENP-P, CENP-Q and CENP-U of humans) [28], none of which is identified in *Drosophila*. Similarly, the human Mis12 complex has been proposed to interact with the CCAN subunit CENP-T [22,23,42]. The latter additionally interacts with the Ndc80 complex, contributing to its recruitment and to a general stabilization of the outer kinetochore [7,21,42,72–76].

We identify two distinct *Drosophila* Mis12 complexes, containing either the Nnf1a or the Nnf1b subunit. Our extensive biochemical and biophysical analyses failed to reveal significant differences in the behaviour of these complexes. In each of the complexes, the Mis12 and Nnf1 subunits (a or b) form a tight dimer and create the binding site for Kmn1^Nsl1^, which in turn creates a binding site for Spc105R^Knl1^. Furthermore, both complexes interact tightly with the N-terminal region of CENP-C and with the C-terminal region of Spc105R^Knl1^, suggesting that they have similar or indistinguishable interaction potentials. However, previous studies demonstrated different developmental expression patterns for Nnf1a and Nnf1b, suggesting the possibility of functional specialization of the two complexes [34,63].

Despite considerable sequence divergence of the DmCENP-C and DmSpc105^Knl1^ binding regions, the interactions they entertain with the Mis12 complex engage topologically equivalent regions of their primary structure (near the N-terminus of CENP-C and the C-terminus of Spc105R^Knl1^). Our mutational analysis identifies the N-terminal region of the Mis12 subunit as a primary determinant of CENP-C binding. An overall conclusion emerging from these studies, therefore, is that kinetochores display considerable evolutionary and structural plasticity. How this plasticity can be accommodated in the structure of the Mis12 complex remains unclear, and our future work will aim to address this urgent question by direct structural analysis.

## 3. Methods

### 3.1. cDNAs and DNA constructs

The cDNA for DmSpc105^1707–1960^ was amplified by the polymerase chain reaction (PCR) from the pOT2 vector containing the full-length DmSpc105R^Knl1^ sequence (isoform A; a generous gift of Christian Lehner's Lab in University of Zurich) and subcloned into the fourth cassette of pST44 [64]. Optimized (for *E. coli*) coding sequences for DmMis12, DmNnf1a, DmNnf1b, DmKmn1 and full-length DmCenp-C were obtained from GeneArt. DmCenp-C fragments were amplified by PCR and subcloned into the pETDuet-MBP8His, a modified version of pETDuet vector (Novagen) generated in house. Sequences encoding variant versions of the DmMis12 complexes were generated in the pST44 system using standard restriction enzyme-based cloning procedures. The QuikChange mutagenesis kit (Agilent Technologies) was used to generate all mutant versions of the plasmids.

### 3.2. Protein expression and purification *Escherichia coli*

BL21(DE3) Rosetta cells were used to express all recombinant proteins. Cells were grown in Terrific broth at 37°C to an OD_600_ of about 0.8. Protein expression was induced by addition of 0.1 mM IPTG at 20°C, and cells were incubated overnight. Cell pellets were resuspended in binding buffer (20 mM Tris/HCl pH 8.0, 300 mM NaCl, 5% (v/v) glycerol, 1 mM EDTA, 1 mM TCEP), lysed by sonication and cleared by centrifugation at 10 000*g* for 30 min. The cleared lysate was purified through a succession of His-Trap HP, HP ResourceQ and Superdex 200 10/300 columns (GE Healthcare).

### 3.3. Analytical size-exclusion chromatography

Analytical size-exclusion chromatography experiments were performed on calibrated Superdex200 5/150 column (GE Healthcare). All samples were eluted under isocratic conditions at 4°C in size-exclusion chromatography buffer (20 mM Tris, 150 mM NaCl, 1 mM TCEP) at a flow rate of 0.2 ml min^−1^. Elution of proteins was monitored at 280 nm. The loading volume for each injection was 50 µl. In order to detect complex formation, proteins were mixed at 1 : 1 (molar ratio) and incubated for 2 h on ice. SDS–PAGE, followed by Coomassie staining, was used to detect proteins.

### 3.4. Negative-stain electron microscopy

The Mis12 complex was diluted to 15 nM for EM grid preparation. About 4 µl of protein sample was adsorbed onto glow-discharged carbon-coated grids for 1 min at 25°C, prior to negative staining with 0.07% uranyl formate (SPI supplies/Structure Probe). Samples were imaged with a JEOL1400 microscope equipped with a LaB6 cathode operating at 120 kV. Images were recorded at low-dose conditions at a magnification of 67 200 on a 4 × 4 k charge-coupled device (CCD) camera (TVIPS GmbH).

### 3.5. Sedimentation velocity analytical ultracentrifugation

Sedimentation velocity experiments were performed in an Optima XL-A analytical ultracentrifuge (Beckman Coulter, Palo Alto, CA) with Epon charcoal-filled double-sector quartz cells and an An-60 Ti rotor (Beckman Coulter). Samples were dialysed against buffer (20 mM Tris pH 8, 0.15 M NaCl and 1 mM TCEP) that was used as a reference. Samples were centrifuged at 42 000 rpm at 20°C, and 500 radial absorbance scans at 280 nm were collected with a time interval of 1 min. The data were analysed using the SEDFIT software [77] in terms of continuous distribution function of sedimentation coefficients (c(S)). The protein partial specific volume was estimated from the amino acid sequence using the program SEDNTERP. Data were plotted using the program GUSSI.

### 3.6. Cross-linking/mass spectrometry

About 0.8 mg ml^−1^ DmMis12a was mixed with 0.6 mM BS2G-H6/D6 (Creative Molecules, www.creativemolecules.com) in a final volume of 50 µl. After incubation for 30 min at 37°C, the reaction was quenched by adding 100 mM ammonium bicarbonate and incubating 15 min at 37°C. Cross-linked proteins were digested, and the cross-linked peptides were enriched and analysed by liquid chromatography coupled to tandem mass spectrometry using a hybrid LTQ-Orbitrap Elite instrument (Thermo Fisher Scientific, Waltham, MA) [66]. Cross-links were identified by the dedicated software xQuest [78]. False discovery rates (FDRs) were estimated using xProphet [78], and results were filtered according to the following parameters: FDR < 0.05, min delta score = 0.90, MS1 tolerance window of −4 to 4 ppm, Id-score > 22. Cross-links were visualized using the xVis web server [79].

## Supplementary Material

Supplemental-Figure-Legends

## Supplementary Material

Figure S1

## Supplementary Material

Figure S2

## Supplementary Material

Figure S3

## Supplementary Material

Figure S4

## Supplementary Material

Figure S5

## Supplementary Material

Figure S6
