# Supplementary material for: Insights from the reconstitution of the divergent outer kinetochore of Drosophila melanogaster
Source: Open Biol. 2016 Feb 24;6(2):150236. doi: 10.1098/rsob.150236 (PMC4772808; doi:10.1098/rsob.150236)
Supplement: Supplemental-Figure-Legends [file rsob150236supp1.docx]

**Supplemental Figure Legends**

**Figure S1 *Additional biochemical analyses***

**A**) Size-exclusion chromatography of binary complexes of Mis12 and Nnf1a or Nnf1b; **B**) Cross-linking of the indicated samples with BS2G (bis[sulfo-succinimidyl]glutarate) leads to the accumulation of high molecular weight species that were subsequently subjected to mass spectrometry analysis; **C**) Cross-linking-mass spectrometry (XL-MS) analysis of the DmMis12b complex. Blue and red lines indicate inter- and intra-molecular cross-links, respectively.

**Figure S2 *Alignments of N-terminal regions of CENP-C***

Initial alignments of CENP-C in Drosophilids, vertebrates, and yeasts were created with PSI BLAST (<http://blast.ncbi.nlm.nih.gov>). A subset of sequences was then imported in program MUSCLE (1) for a refined alignment and visualized in Jalview (2).

**Figure S3 *Additional size-exclusion chromatography analyses***

**A**-**F**) Size-exclusion chromatography analyses of the indicated species.

**Figure S4 *Additional biochemical analyses on the Mis12b:CENP-C complex***

**A**) Sedimentation velocity absorbance profiles of the DmMis12a and DmMis12b complexes, with residuals of the fit showing the deviation of the c(*S*) model from the observed signals; the best-fit continuous-size c(*S*) distribution of the DmMis12a and DmMis12b complexes is shown on the bottom panel; **B**) Cross-linking-mass spectrometry (XL-MS) analysis of the DmMis12b:CENP-C^1-105^ complex. Blue and red lines indicate inter- and intra-molecular cross-links, respectively.

**Figure S5 *Additional size-exclusion chromatography analyses of mutant Mis12 complexes***

**A**) Size-exclusion chromatography profile of a mutant of the Mis12a complex in which 15 residues at the N-terminus of the Mis12 subunit were deleted (indicated as Mis12^ΔN15^). Note the complete lack of interaction with CENP-C^1-105^. **B**) Lack of CENP-C binding by the mutant Mis12b complex expressing Phe to Asp (F to D) mutations on residues 12, 13, and 15 of the Mis12 subunits.

**Figure S6 *Analytical ultracentrifugation analyses of the complexes of Mis12a and Mis12b with Spc105R***

**A-B**) Sedimentation velocity absorbance profiles of the DmMis12a and DmMis12b complexes with Spc105R^1707-1960^, with residuals of the fit showing the deviation of the c(*S*) model from the observed signals; the best-fit continuous-size c(*S*) distribution of the DmMis12a and DmMis12b complexes is shown on the bottom panel.

**Supplemental References**

1. Edgar RC. MUSCLE: multiple sequence alignment with high accuracy and high throughput. Nucleic Acids Res. 2004;32(5):1792-7.

2. Waterhouse AM, Procter JB, Martin DM, Clamp M, Barton GJ. Jalview Version 2--a multiple sequence alignment editor and analysis workbench. Bioinformatics. 2009;25(9):1189-91.
